# Effect of ZnO surface morphology on its electrochemical performance

**DOI:** 10.1039/d1ra03653j

**Published:** 2021-07-01

**Authors:** Hajar Ghannam, J. P. B. Silva, Adil Chahboun

**Affiliations:** Université Abdelmalek Essaadi, FST Tanger, Laboratoire Couches Minces et Nanomatériaux (CMN) 90000 Tanger Morocco ghannam11hajar@gmail.com; Centro de Fìsica das Universidades do Minho e do Porto (CF-UM-UP), Campus de Gualtar 4710-057 Braga Portugal

## Abstract

The purpose of this paper is to bridge the gap between ZnO surface morphology and its electrochemical performance. For this reason, ZnO nanowires (NWs) of different length were synthesized using an electrochemical method. Then, the electrochemical performance of the synthesized ZnO surfaces was studied using cyclic voltammetry and electrochemical impedance spectroscopy. The electrochemical analysis results revealed that the increase of ZnO NW length contributes to the retrogression of electrochemical performance. Indeed, the electrochemical performance is mainly related to the wettability behavior of the ZnO nanowire surfaces. When the ZnO NWs length increases, the surface become more hydrophobic, therefore, charge transfers between the electrode/electrolyte decrease. To improve the electrochemical performance of ZnO, we propose a new strategy combining NWs and microsheets (μSs) for further improving the morphology. Finally, the surfaces based on the double structure of ZnO provide good propagation of charge at the surface, good transfer in the electrode, good stability, and excellent scanning ability. In the present work we intend to pave the way for achieving high electrochemical performance ZnO-based layers.

## Abstract 2

The purpose of this paper is to bridge the gap between ZnO surface morphology and its electrochemical performance.

## Graphical abstract

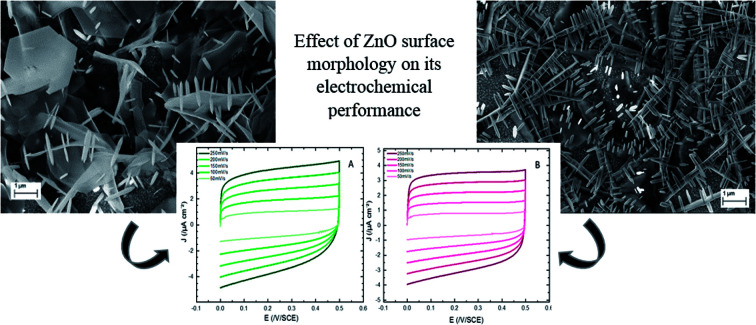

## 1 Introduction

Zinc oxide (ZnO) is the subject of focused research due to its unique physical and chemical properties allowing it to be a multifunctional material.^1^ In materials science, ZnO is classified as a semiconductor in group II–VI and it is characterized by a wide band-gap (3.37 eV), broad range of radiation absorption, and high chemical, thermal and mechanical stability at room temperature.^2,3^ ZnO properties are suitable for various applications such as solar cells, light-emitting diodes, electrochemical sensors, supercapacitors, photocatalysts, transparent UV resistant coatings and self-cleaning glass.^4–10^ On the other hand, ZnO provides one of greatest ranges of forms: it can occurs in one, two and three-dimensional forms. The largest group of ZnO forms is one dimension; rods, wires, ribbons, belts, tubes, and helixes.^11–13^ For two-dimensional forms of ZnO, we find pellets and sheets,^14,15^ while spheres and flowers are examples of three-dimensional forms of ZnO.^16–18^ In general, several synthetic methods are used to utilise ZnO in different forms, such as microfluidic, hydrothermal, electrochemical, and sol–gel methods.^19–21^ However, the most used method is the electrochemical one, where different forms of ZnO are obtained by varying the conditions.^19,22,23^

Several studies have been carried out with the aim of achieving the high potential performance of ZnO. The significant influence of ZnO morphology on electrochemical performance has been shown in two of the main applications; various sensors for electrochemical platforms and energy storage.^24–28^ Indeed, it has been reported using electrochemical characterization that ZnO is required for the advancement of economical sensors with good sensitivity, stability, and rapid response.^24,25^ On the other hand, ZnO has also been widely studied as an electrode in supercapacitors thanks to its pseudocapacitance rising from charge transfer between the electrode/electrolyte. Sasirekha *et al.*^29^ studied ZnO and carbon coated ZnO nanoparticles as supercapacitor materials, and found that the carbon coated ZnO nanoparticle electrode yields a specific cell capacitance of 92 F g^−1^ at the specific current of 2.5 A g^−1^ for symmetric supercapacitor devices. The effect of ZnO contribution on supercapacitor performance has also been reported by Zeng *et al.*^30^ They demonstrated that ZnO nanowires/graphene oxide is a promising candidate for supercapacitors. He *et al.*^31^ studied the electrochemical performance of two main ZnO structures, developing different surface areas where the surface area of ZnO nanocones is superior to nanowires; they found that the ZnO nanocones have a high specific capacitance, about 378.5 F g^−1^ at a scan rate of 20 mV s^−1^, almost twice than of ZnO nanowires.

Several researchers have been presented a correlation between the chemical and physical nature of surfaces with their electrochemical performance.^32–34^ The purpose of this paper is to highlight the effect of ZnO morphology on its electrochemical performance. In this work, ZnO nano/microstructured thin films were synthesized using an electrochemical method. The crystal structure of the ZnO surface was investigated by X-ray diffraction (XRD). Morphology and chemical elements of the synthesized ZnO thin films were characterized by scanning electron microscopy combined with energy dispersive spectroscopy (FEG-SEM). The effect of surface morphology on capacitive performance was reported through electrochemical analysis by cyclic voltammetry (CV) and electrochemical impedance spectroscopy (EIS). Moreover, wettability of the surfaces was quantified by measuring the contact angles.

## 2 Experimental section

ZnO was electrodeposited using a conventional cell of three electrodes connected to a potentiostat/galvanostat (Gamry 600+): counter electrode “platinum grid”, reference electrode “saturated calomel electrode” and working electrode “Solems fluorine doped tin oxide (FTO) glass, thickness 80 nm”. The FTO glass was cleaned before use in an ultrasonic bath with acetone and ethanol, respectively, for 10 min, and then it was rinsed with distilled water. These three electrodes were immersed vertically in an aqueous electrolyte composed of ionic species necessary for ZnO formation (ZnCl_2_: MERCK, purity > 95%, KCl: VWR International, purity 99.0100.5%, Al(NO_3_)_3_: Sigma-Aldrich, purity > 99.99%, and dioxygen). The homogeneity of the aqueous electrolyte was ensured during the electrodeposition time through a magnetic stirrer, where the temperature was kept thanks to a cryostat bath.

ZnO NWs were synthesized from an O_2_ saturated aqueous electrolyte of 0.2 mM of ZnCl_2_ and 0.5 M of KCl, with potential and temperature parameters being held constant at −1 V/SCE and 80 °C, and by varying the electrodeposition time between 1000 s and 7000 s.

ZnO microsheets (μSs) were synthesized from an aqueous solution saturated by bubbling oxygen, composed of 2.5 mM of ZnCl_2_ 0.5 mM of Al(NO_3_)_3_ and 0.1 M of KCl, at a constant temperature (80 °C) and potential (−1 V/SCE) for electrodeposition time of 1000 s and 2000 s.

The mass (*m*) of ZnO formed on the FTO surface was estimated from Faraday’s law (eqn (1)), where the total electric charge (*Q* in coulombs) is the result of chronoamperometric curve integration, *F* (= 96 500 C mol^−1^) is the Faraday constant, *z* is the number of transferred electrons per ion which is equal to 2, and *M* is the molar mass of ZnO (81.38 g mol^−1^).1
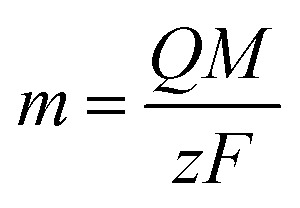


The crystal structure of the ZnO surfaces was investigated using an Empyrean Panalytical X-ray diffractometer. The morphology and elemental analyses of thin films of ZnO were characterized using a field emission scanning electron microscope and energy dispersive spectroscopy (EDS) system (FEG-SEM) ULTRA 55 ZEISS. Moreover, the 3D and profile roughness curves of ZnO are depicted through 2D SEM images. The electrochemical analysis, CV and EIS, were performed using a potentiostat/galvanostat (Gamry 600+). The wettability of ZnO surfaces was quantified through the contact angle measurement using a drop shape analyzer, Krüss DSA100.

## 3 Results and discussion

**Fig. 1:**
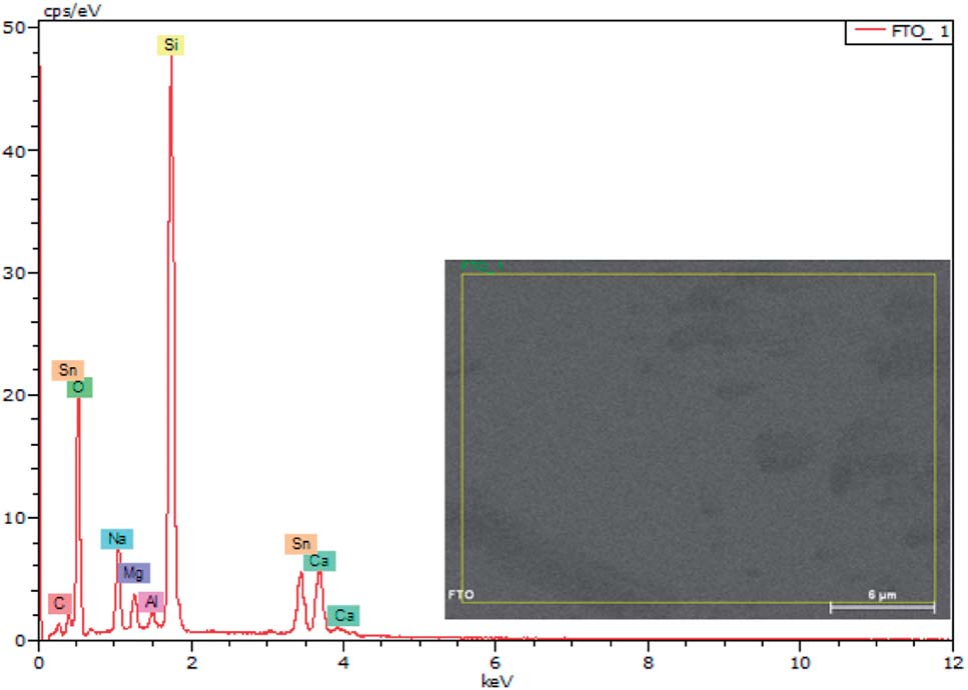
EDX spectrum of FTO glass.

**Fig. 2:**
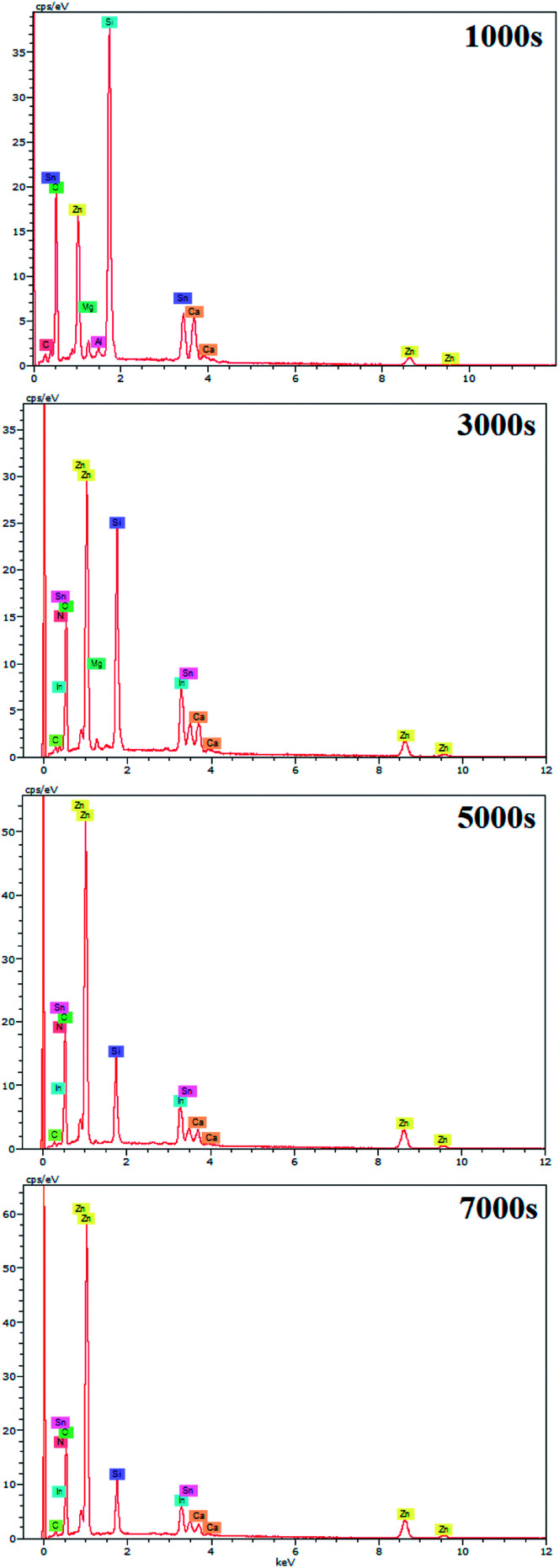
EDX spectra of ZnO nanowires obtained after different amounts of synthesis time.

**Fig. 3:**
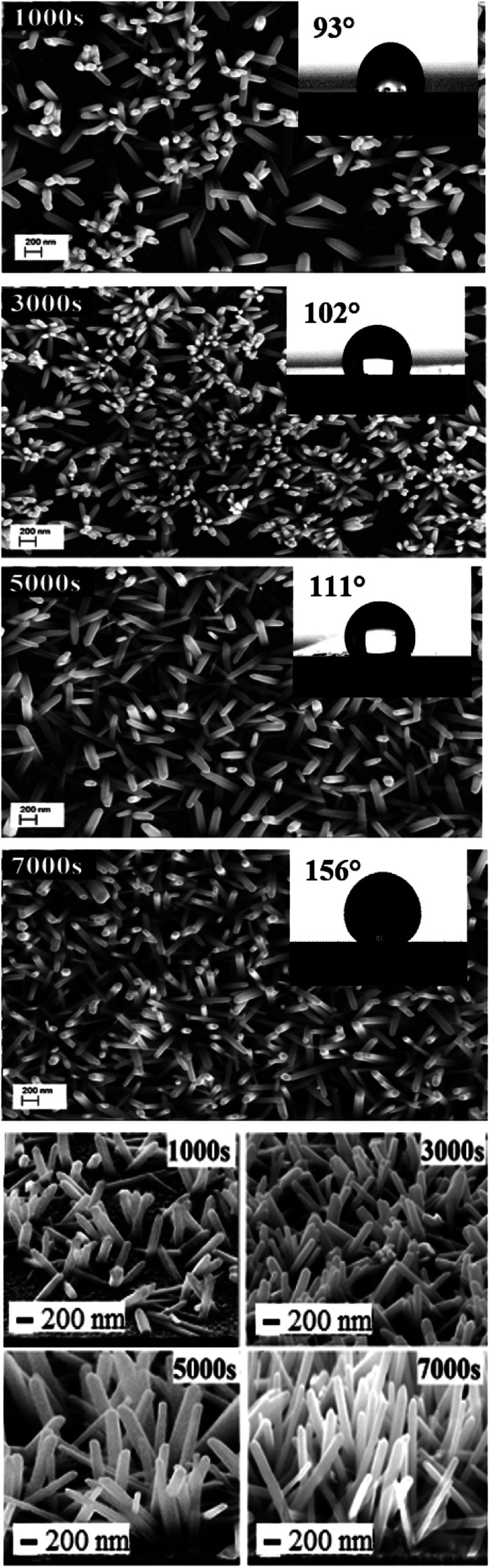
Plan and cross-sectional views of ZnO NW SEM images synthesized after different amounts of time, *t* = 1000 s, 3000 s, 5000 s, and 7000 s ([ZnCl_2_] = 0.2 mM, [KCl] = 0.5 M, *T* = 80 °C and *E* = −1 V/SCE), and their corresponding wettability behavior.

EDX results show the elemental chemistry of the synthesized ZnO thin films. Other than the electrodeposited ZnO, we clearly see the elements of our substrate consisting of glass on which FTO is deposited (Fig. 1). The glass is mainly made of silicon dioxide (SiO_2_) and sodium oxide (Na_2_O). The featured lines peaking at (0.592, 0.691), (1.012, 1.041), 1.739, and 8.630 keV are assigned to the (L line of Sn, K line of O), (K line of Zn, K line of Na), K line of Si, and K line of Zn elements, respectively. Moreover, less intense lines are observed for the K line of Mg and Al elements at 0.637, and 1.486 keV, respectively. These elements originated from the glass additives. The EDX spectra of the synthesized ZnO surfaces show that the Zn and O lines become more intense when the electrodeposition time of ZnO increases (Fig. 2). The SEM images depict the morphology of ZnO nanowires growth on FTO at different electrodeposition times (Fig. 3). As is shown, the electrodeposition time mainly influences the height of ZnO NWs and therefore, the increase in electrodeposition times gives rise to long NWs.

**Fig. 4:**
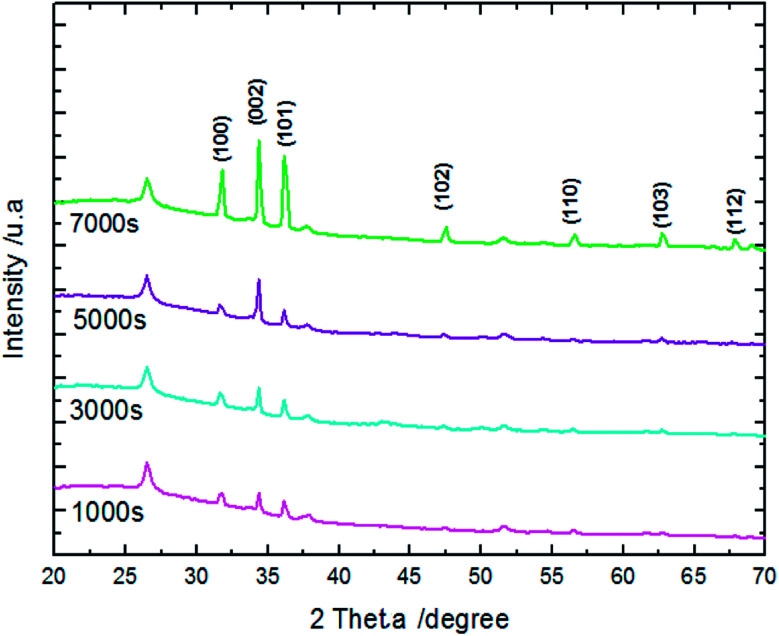
XRD patterns of ZnO nanowires synthesized after different amounts of synthesis time.

The XRD patterns reveal that ZnO crystallizes with the wurtzite crystal structure and in Fig. 4 the (100), (002), (101), (102), (110), (103), (200) and (112) crystal planes located at 2θ values of 31.8°, 34.4°, 36.3°, 47.5°, 56.6°, 62.9°, 66.4°, and 68.0°, respectively, are identified. Moreover, the peaks that appear at 2θ values of 27° and 52° correspond to the FTO crystal structure. Fig. 4 shows that the ZnO structure grows along the direction of the polar (002) plane, preferentially. As the time increases, it is possible to observe an increase in the intensity of the XRD peak, which can be associated with the increase of ZnO NW length along the *c*-axis.^19^ ZnO growth along the *c* axis is mainly related to ZnO wurtzite structure polarity. Indeed, the absence of a symmetry center in the ZnO wurtzite structure along the *c*-axis leads to the formation of ZnO instable polar facets. These instable facets, (0001) and (0001̄), are more reactive *vis-à-vis* the growth of ZnO.^19^ However, slim NWs of ZnO mainly occur thanks to the large difference of diffusion coefficient between Zn^2+^ and O_2_, along with the low concentrations of Zn^2+^ precursor in the solution (between 5 × 10^−5^ and 0.02 M). Where, the Zn^2+^ ions present in low concentrations in the solution are quickly consumed in reactions between OH^−^ ions adsorbed in the polar facets of ZnO.^22,35^ Furthermore, the variation in ZnO NW morphology over time match well with the mass of ZnO which increases from 151 μg at 1000 s to 775 μg at 7000 s (Table 1).

**Table 1:** The estimated mass of ZnO deposited on the cathode electrode obtained from chrono-amperometric curves for different amounts of electrosynthesis time

Sample	1000 s	3000 s	5000 s	7000 s
Charge density (C cm^−2^)	0.36	1.1	1.47	1.84
Mass of ZnO (μg)	151	463	619	775

**Fig. 5:**
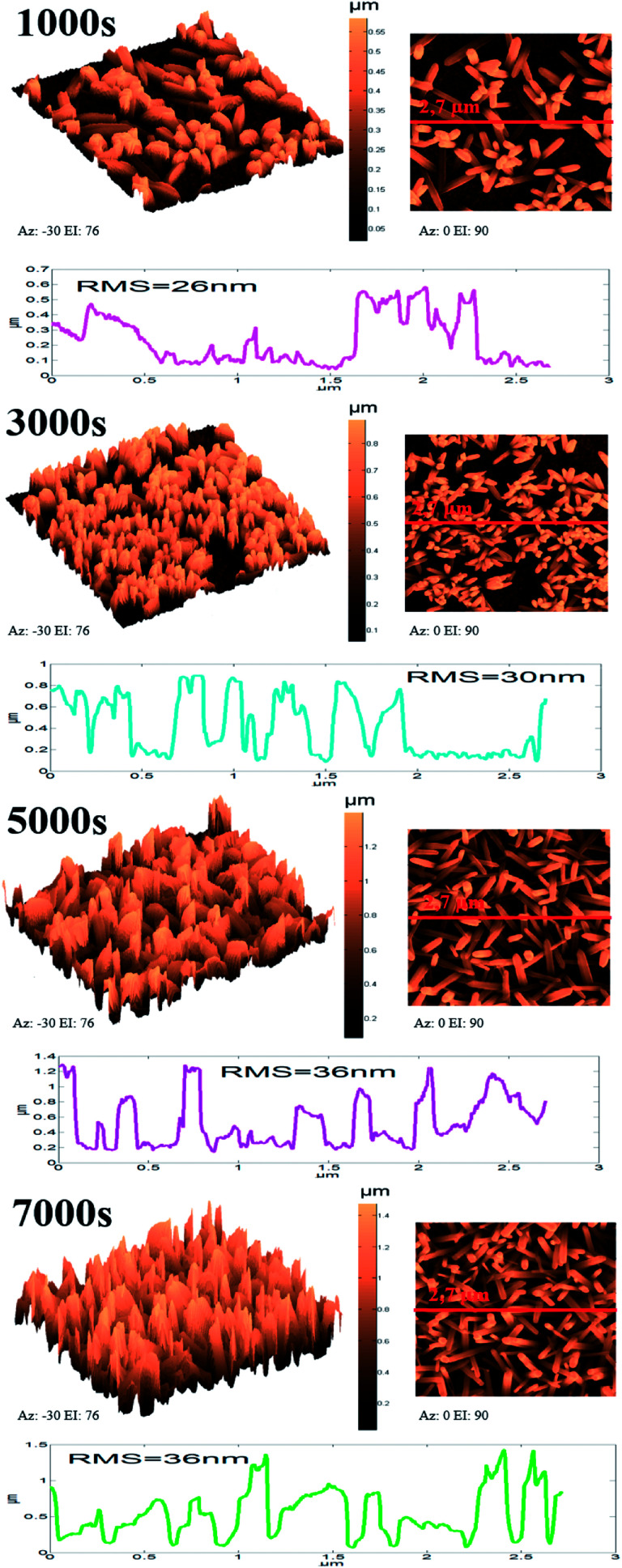
3D, 2D, and profile roughness curves of ZnO nanowires obtained after different amounts of synthesis time.

ZnO NW surface topographies are shown in Fig. 5. With increasing electrodeposition time, the density of ZnO become more accented, and produces a distinctly different looking surface with sharp structures and deep valleys. Moreover, a general trend of increasing roughness curve, with increasing electrodeposition time, is observed and further confirmed by the root-mean-square (RMS) values, which increase from 26 to 36 nm. Regarding the wettability behavior of ZnO NW surfaces, we find that the hydrophobicity of the ZnO surface increases with the length of ZnO NWs. The hydrophobic behaviour of the samples is related to the preponderance of ZnO non-polar facets. In fact, it was reported that the ZnO non-polar facets have the ability to repulse water molecules, producing a hydrophobic contact.^36^ Also, it was been reported that the roughness increases the wetting quality; the surfaces become more hydrophobic when the roughness of hydrophobic surfaces increases.^37^ When the roughness of hydrophobic surfaces increases the liquid cannot penetrate into crevices due to the appearance of air pockets, that have a contact angle of 180° ‘Fakir state’. The contact angle evolution on ZnO NW surfaces (93, 102, 111, and 156°, for example 1000 s, 3000 s, 5000 s, and 7000 s, respectively) agree with the different phenomena of liquid penetration observed on hydrophobic surfaces.^38^

**Fig. 6:**
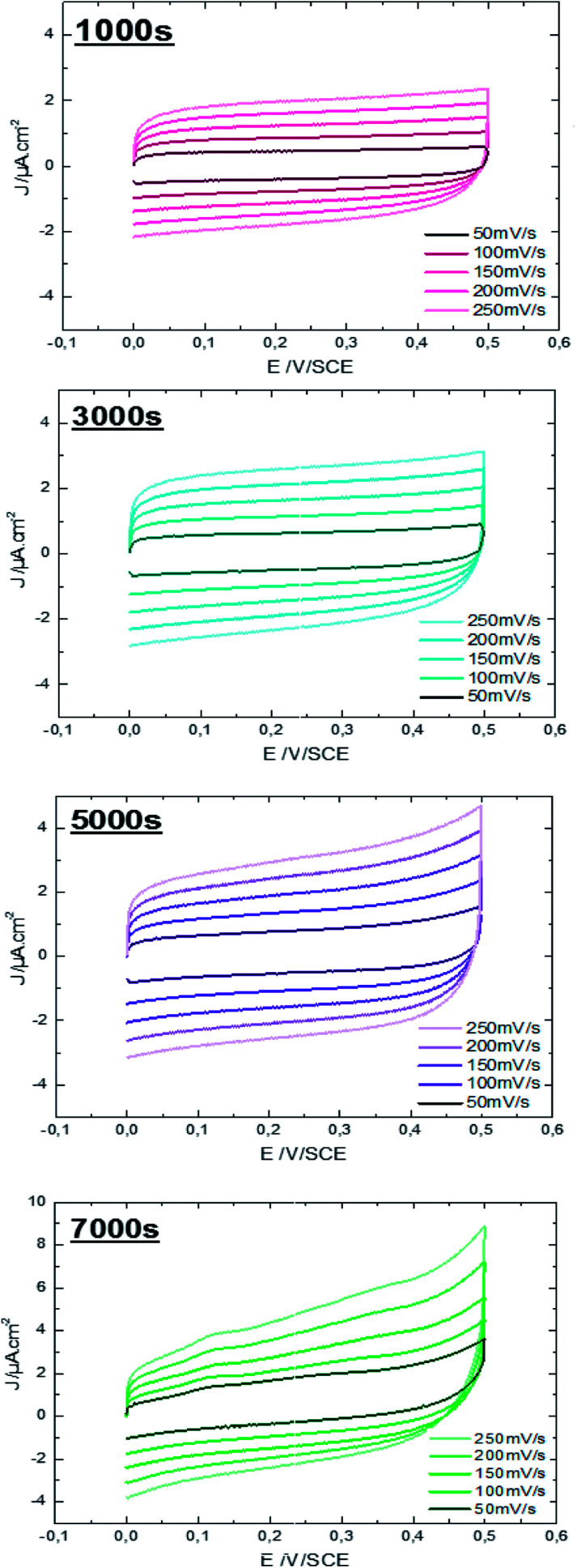
Cyclic voltammetry performance of ZnO NWs (*t* = 1000 s, 3000 s, 5000 s, and 7000 s) at different scan rates in 0.1 M aqueous KCl solution.

Characterization of ZnO surfaces is achieved by working in the absence of faradic reactions and in conditions of ideal polarizability. The effect of ZnO morphology on capacity performance is elucidated using CV measurements. Fig. 6 shows CV responses of ZnO NW surfaces, deposited at different times, running at different scan rates within the window of potentials of material stability (potential ranging from 0 to 0.5 V) in an aqueous electrolyte of 0.1 M KCl at ambient temperature (25 °C).

It is observed, that the integral area of the CV loop increases with the electrodeposition time of ZnO NWs (Fig. 6). In general, a large specific surface area promotes the transfer of electrons between the charge collector and the active material. Therefore, more charges to store and transfer in the electrodes are provided. Moreover, by comparing the shape of the CV loops of ZnO NWs synthesized at different times, we find that the shapes of CV curves considerably distort when the synthesis time increases. For synthesis time lower or equal to 3000 s, the shape of the CV curves are rectangular. This shape indicates the good propagation of charge at the surface of ZnO NWs. However, for synthesis times of 5000 s, the shape of the CV loop weakly distorts. The distortion of the CV loops is more pronounced for the NWs synthesized at 7000 s. In fact, the distortion of the CV loop is mainly caused by the increased height and density of hydrophobic ZnO NWs which probably leads to formation of the air pockets at the electrode surface enhancing the good propagation of electrolytes. Furthermore, for all synthesized samples, the CV curves retain the same shape for different scan rates. This indicates the good stability of the electrode surface. Besides, their density of electrical current increases with increasing scan rates confirming the excellent scanning ability of the samples.

**Fig. 7:**
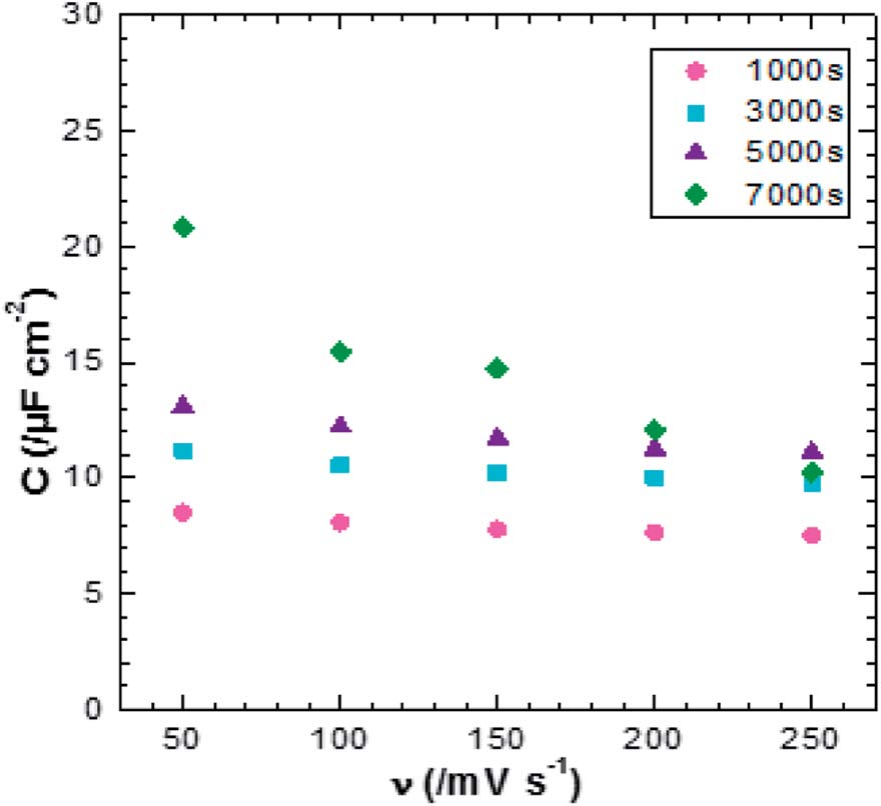
Plot of scan rate verse specific capacitance of ZnO NWs after different amounts of synthesis time.

The specific capacitance is calculated from CV curves for different scan rates using the following formula:2
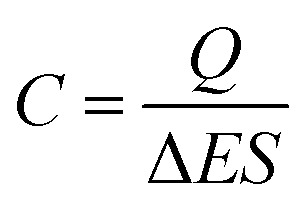
where, *Q* is the average charge during the charge and discharging processes, Δ*E* is the potential window, and *S* is the electrode geometrical surface. The scan rate *versus* specific capacitance of ZnO NWs synthesized over different times is shown in Fig. 7. In general, the concentration of conductive species (electrons/holes) of semiconductor materials is much smaller than that in solution. In this case, the space charge is redistributed at a larger distance – 10–100 nm – in the semiconductor than that in solution, leading to capacitance reduction of the semiconductor electrode.^39,40^ Indeed, it has been reported that for ZnO smooth thin films, the specific capacitance value is very small, about 5.6 μF cm^−2^ at a scan rate of 100 mV s^−1^.^6,41^ In our case, the values of specific capacitance for ZnO NW surfaces are varied from 7 up to 16 μF cm^−2^ at a scan rate of 100 mV s^−1^. Whilst these values increase when the scan rate decreases. The increase of specific capacitance values can be justified by the increasing participation of the active surface area at low scan rates. However, the specific capacitance values as a function of scan rate does not match well with ZnO surface morphology. We find that for relatively high scan rates, <150 mV s^−1^, the morphology of ZnO does not significantly affect the specific capacitance values, where the specific capacitance values are very close, ranging between 7 and 11 μF cm^−2^ for a scan rate of 250 mV s^−1^. Also, we find that for a scan rate of 250 mV s^−1^, the specific capacitance of the sample after 7000 s is lower than of the sample after 5000 s, despite its large specific surface area. This can be justified considering the wettability behavior of the sample. Indeed, when the ZnO nanowire morphology increases, the surface become more hydrophobic (the contact angle increases from 93° for the sample after 1000 s to 156° for sample after 7000 s). Therefore, the charge transfer between electrode/electrolyte decreases and thus the capacitance values decreases.

**Fig. 8:**
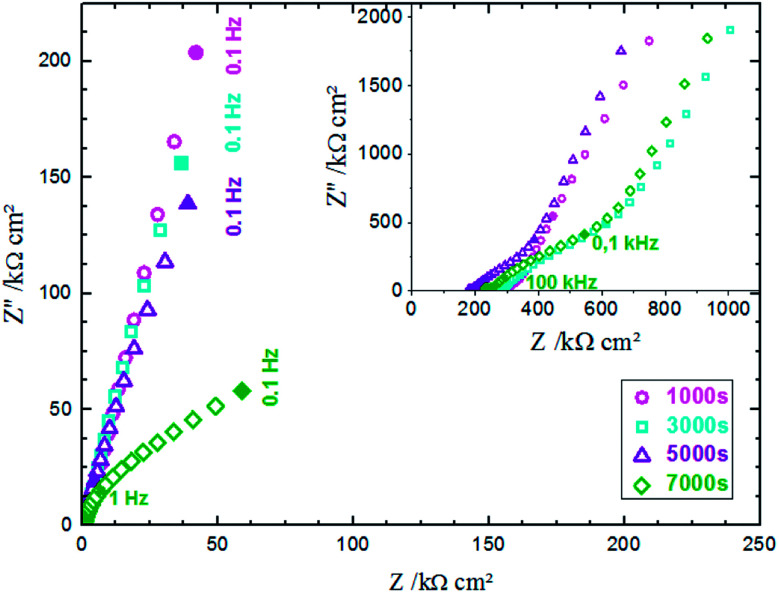
Electrochemical impedance spectra of ZnO NWs synthesized after different amounts of time in 0.1 M KCl solution.

EIS was performed in an aqueous electrolyte solution of 0.1 M KCl at ambient temperature (25 °C). The Nyquist plot is commonly used to represent the impedance measurements. Fig. 8 presents the Nyquist plots of ZnO NWs synthesized after different amounts of time. The results show that the resistances of the electrolyte solution determined by the real axis intercept at high frequency are in the order 1000 s < 3000 s < 5000 s < 7000 s. Indeed, electrolyte resistance is inversely proportional to real electrode surface. The increase of the electrolyte resistance values with ZnO nanowire length is due to the increase of hydrophobic behaviour of the surface, which reduces the contact between the electrode and electrolyte. However, there is an absence of relationship between series resistance and capacitive performance due to functionalization of the ZnO surfaces.^42^

The modification of ZnO nanowires leads to modification of liquid penetration and thus the surface wettability. For the highly ramified surfaces, the liquid is less propagated into the surface asperities and this leads to the retrogression of electrochemical performance. Since the surface of ZnO nanowires synthesized at 1000 s is more stable, we proposed to improve the electrochemical performance of ZnO NWs by combining them with ZnO μSs. Indeed, μSs of ZnO are know to be more wettable because of the dominance of the polar facets.

**Fig. 9:**
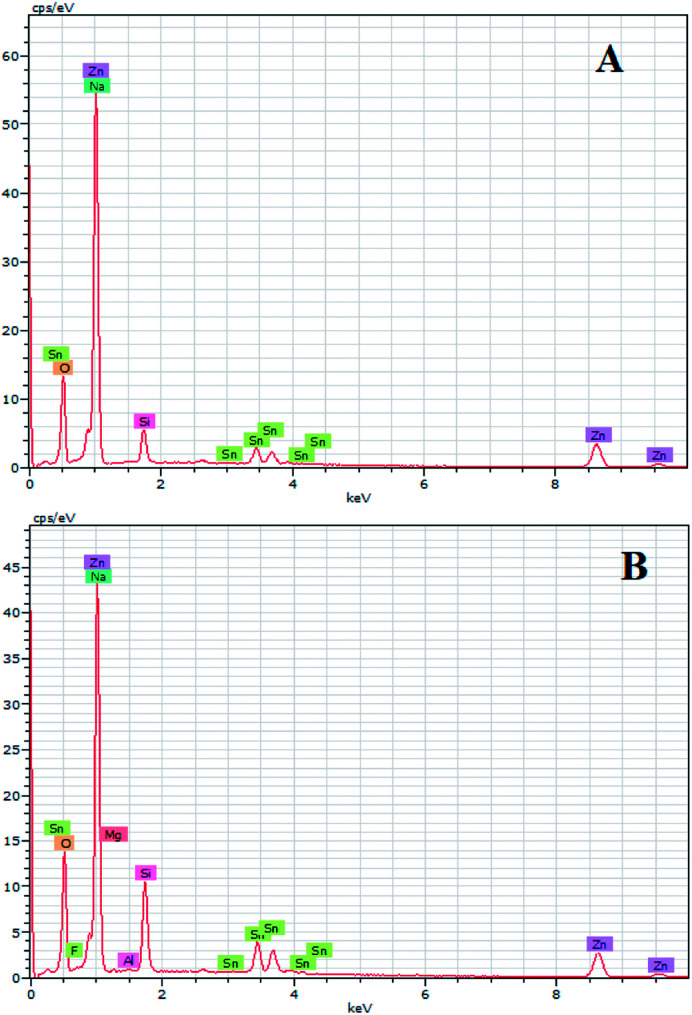
EDX spectra of sample A and B.

**Fig. 10:**
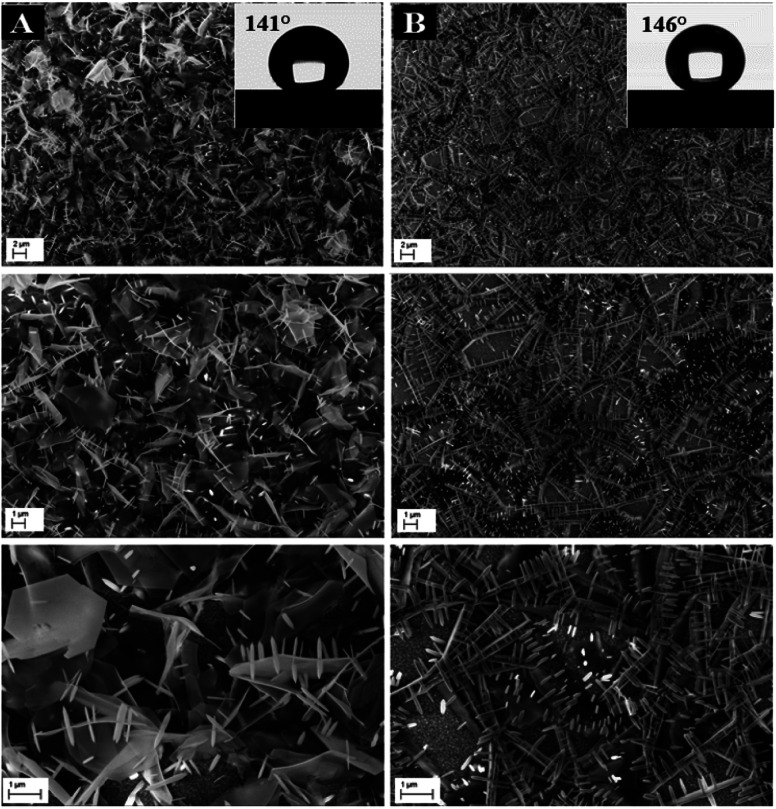
SEM images of ZnO with double scale roughness. Sample A (microsheets (*t* = 2000 s) + nanowires (*t* = 1000 s)) and B (microsheets (*t* = 1000 s) + nanowires (*t* = 1000 s)) and their corresponding wettability behavior.

ZnO with two structures was successfully synthesized by combining ZnO NWs and ZnO μSs. The ZnO μSs of sample A are more dense than sample B. However, the nanowires are the same for both samples (Fig. 10). Wettability results show that the ZnO surface based on the double structure exhibits hydrophobic behavior with contact angles of 141 and 146° for sample A and B, respectively (Fig. 10). Fig. 9 shows the EDX elemental analyses of sample A and B. The EDX spectra show the chemical elements of the FTO substrate depicted in Fig. 1, and also the elements of ZnO electrodeposited. Indeed, we observe that the intensity of the peaks of the chemical elements of ZnO increase with the mass of ZnO which is equal to 1560 μg for sample A and 657 μg for sample B (Table 2).

**Table 2:** The estimated mass of ZnO deposited on the cathode electrode obtained from chrono-amperometric curves after different amounts of electrosynthesis time

Sample	A	B
Charge density (C cm^−2^)	3.69	1.56
Mass of ZnO (μg)	1560	657

**Fig. 11:**
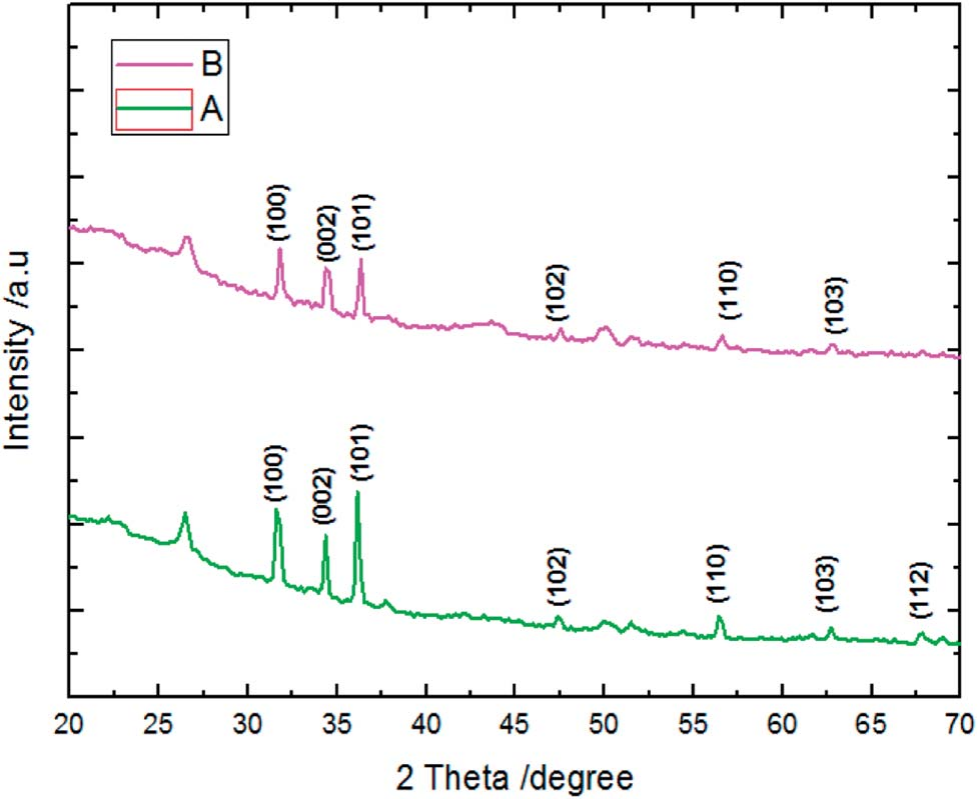
XRD patterns of samples A and B.

The conditions of μSs electrodeposition used in this work were reported for the first time by Ghannam *et al.*^38^ For these conditions, the μSs growth is considered much faster than the others previously used.^43–45^ Indeed, μSs are the result of adsorption of Cl^−^ ions – provided both from ZnCl_2_ and KCl – on polar facets of ZnO enhancing the natural growth along the (0002) plane. Whilst the growth along non polar facets of ZnO arises thanks to OH^−^ ions – provided from nitrate and molecular oxygen – which react with Zn^2+^ ions on the (101̄0) plane. From the XRD results of samples A and B, we find that the structure of ZnO crystallized in wurtzite presented a preferential growth along (100) and (101) planes, thus orientation along the *a*-axis is ascribed to microsheet morphology (Fig. 11).

**Fig. 12:**
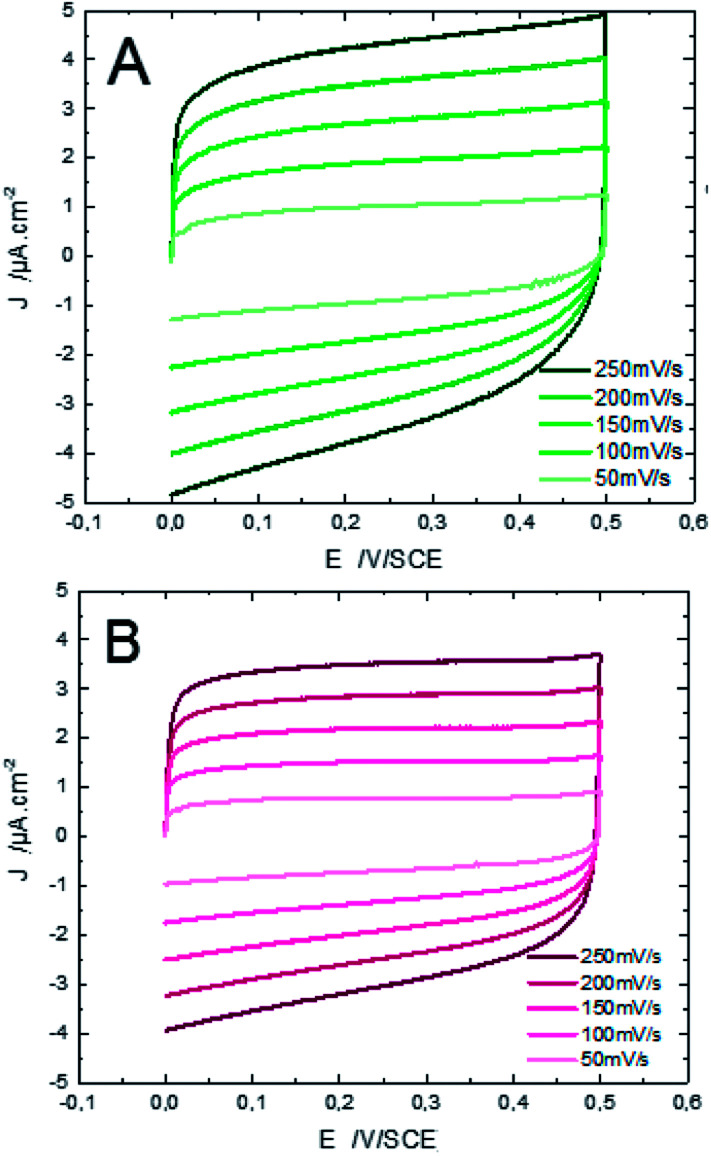
Cyclic voltammetry performance of ZnO with double scale roughness at different scan rates in a 0.1 M aqueous KCl solution. Samples A (microsheets (*t* = 2000 s) + nanowires (*t* = 1000 s)) and B (microsheets (*t* = 1000 s) + nanowires (*t* = 1000 s)).

**Fig. 13:**
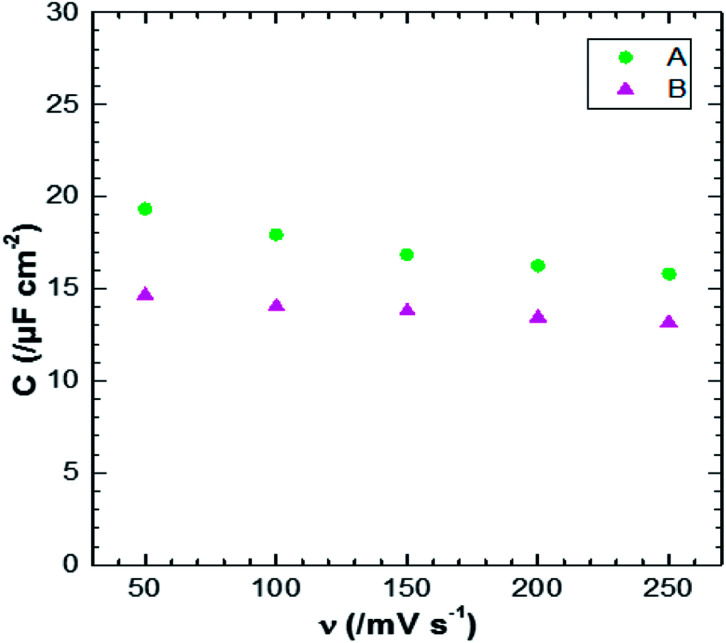
Plot of scan rate *versus* specific capacitance for samples A and B of ZnO double structure.

**Fig. 14:**
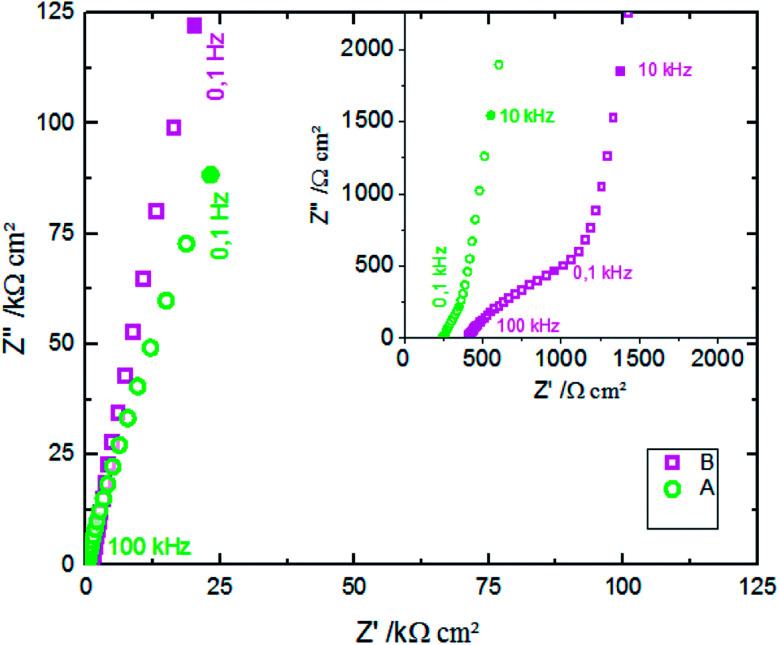
Electrochemical impedance spectra of the samples A and B in 0.1 M KCl solution.

Fig. 12 shows the CV responses of samples A and B. The CV loops of samples A and B are rectangular, and they retain the same shape for different scan rates which indicates that these surfaces exhibit excellent scanning ability and a good propagation of charge at their surfaces. In fact, by using the double structure of ZnO, we avoid the CV curves distortion appeared from the NWs surface. Regarding the specific capacitance values, Khalil *et al.*^41^ have reported that the specific capacitance of a smooth thin surface of ZnO is about 5.5 μF cm^−2^ at a scan rate of 200 mV s^−1^. However, using the double structure of ZnO, the specific capacitance reached 16.5 μF cm^−2^ at a scan rate of 200 mV s^−1^ (Fig. 13). Whilst, the specific capacitance is about 12 μF cm^−2^ at a scan rate of 200 mV s^−1^ for the longer nanowires of ZnO – sample after 7000 s (Fig. 7). Fig. 14 presents the Nyquist plots of samples A and B. The results show that the resistance of the electrolyte solution of sample B is greater than that of the sample A. This can be justified by the contact angle value which is about 141° for sample A and 146° for sample B. Through electrochemical analysis results of samples A and B, we find that the ZnO surfaces with the double structure seem to lead to more interesting thin films for electrochemical applications.

## 4 Conclusions

In this work, the electrochemical performance of ZnO NWs was tuned using the deposition time. By increasing the time, the ZnO surface area and length were increased and this leads to the retrogression of electrochemical performance due to the hydrophobic behavior of the surfaces. In addition, we have shown that the electrochemical performance can be improved through the combination of ZnO NWs with μSs. In this case, the specific capacitance was increased by 35%, while maintaining the stability of the surface of electrode. This work shows that the association of μSs of ZnO with NWs is a promising strategy to enhance the electrochemical performance of ZnO-based capacitors.

## Conflicts of interest

There are no conflicts to declare.

## Supplementary Material
